# Validation of the Malay Version of the Caregiver Quality of Life Index–Cancer (MCQoL) among caregivers of cancer patients in Malaysia

**DOI:** 10.1371/journal.pone.0335681

**Published:** 2026-01-05

**Authors:** Aisyah Ali, Hui Li Lim, Ruzaini Zulhusni Puslan, Nurul Huda Razali, Chun Sen Lim

**Affiliations:** 1 Clinical Research Centre, Hospital Sultan Ismail, Institute for Clinical Research, National Institute of Health, Ministry of Health, Johor, Malaysia; 2 Centre for Clinical Care and Outcomes Research, Institute for Clinical Research, National Institute of Health, Ministry of Health, Selangor, Malaysia; 3 Mathvision Enrichment Centre Pte Ltd., Singapore, Singapore; 4 Oncology Department, Hospital Sultan Ismail, Ministry of Health, Johor, Malaysia; Ajman University, UNITED ARAB EMIRATES

## Abstract

Cancer caregivers frequently experience significant psychological distress as they manage the increasing treatment burden of cancer patients, which adversely impacts their quality of life. In Malaysia, there has been a notable absence of disease-specific instruments to measure cancer caregivers’ quality of life. Our study aimed to validate the Malay version of the Caregiver Quality of Life (MCQoL) scale among Malaysian cancer caregivers. This cross-sectional study was conducted from 01-09-2022 to 31-07-2023, involving 310 cancer caregivers from a cancer centre in southern Peninsular Malaysia. The MCQoL scale comprises 35 items across four domains: burden, positive adaptation, disruptiveness, and financial concern. Construct validity was assessed using Exploratory Factor Analysis (EFA) and Confirmatory Factor Analysis (CFA). Meanwhile, internal consistency and test-retest reliability were determined using Cronbach’s alpha coefficient and intra-class correlation respectively. The study population were predominantly female (62.80%), married (68.30%) and Malay ethnicity (70.60%), with a mean age of 44.00 (± 14.22) years. The mean MCQoL score was 59.32 (±20.96), with higher scores indicating poorer quality of life. EFA led to the removal of three problematic items, resulting in a modified scale with excellent sampling adequacy (KMO = 0.891, p < 0.001) and a total variance explained of 53.81%. The final CFA supported a four-factor model with an acceptable fit (RMSEA = 0.06, CFI (Comparative Fit Index) = 0.90, Chisq/df ratio $\chi^{2}$/df = 2.32, Tucker-Lewis Index (TLI) = 0.89) and strong internal consistency (Cronbach’s alpha = 0.879). The MCQoL scale demonstrated adequate validity and reliability for assessing quality of life of cancer caregivers in Malaysia. This tool can be implemented within local oncology services to systematically assess and monitor caregiver quality of life, enabling healthcare teams to identify specific areas of need or distress and there by tailor interventions, provide targeted support, and improve overall caregiver well-being.

## Introduction

Cancer is one of the leading causes of mortality and remains a significant barrier to increasing life expectancy worldwide [1]. According to estimates from the International Agency for Research on Cancer, World Health Organization (WHO), approximately 19.3 million new cancer cases were reported in 2020 [1]. By 2040, it is projected that 28.4 million new cancer cases will occur, with countries that have a high Human Development Index (HDI) expected to experience the most significant increase in incidence [1]. Malaysia is classified as a high-HDI country, with 115,238 new cancer cases recorded between 2012 and 2016 [2]. Of these, 40.9% were detected at advanced stages, with the Johor state reporting the highest incidence rate [2].

Cancer is a chronic condition that progresses over the course of a patient’s life [3], and has become a concern for the entire family [4]. Cancer care has gradually shifted toward home care, with family members taking on the role of primary carers for patients [4]. Their responsibilities vary and may include assisting with the patient’s personal needs, maintaining cleanliness, managing finances, arranging care and services, organizing transportation, addressing problems and offering emotional support [5]. While providing cancer care can be a meaningful and fulfilling experience, caregivers often face significant challenges. These include conflicts between their social roles, limitations on their personal activities, strains in marital and family relationships and a decline in their physical health [6–8]. Such challenges can lead to substantial psychological effects [9] and in more severe cases, impair the caregivers’ overall quality of life (QOL) [4].

This concerning for caregivers of patients in advanced stages of cancer, as they are at an even higher risk of experiencing detrimental side effects [10,11]. The prolonged duration of illness and treatment, repeated phases of illness and hospital admissions, uncertain prognosis and increasing side effects from various treatment options further exacerbate caregivers’ burden [12,13]. The decline in QOL among cancer caregivers is often more pronounced in Asian culture, where filial virtue [14] and societal norms compel family members to take on the role of primary caregivers [15]. Notably, these negative impacts on caregivers can create a ripple effect, ultimately affecting the quality of care provided to cancer patients [16]. Therefore, it is of pressing need to assess the level and deficits in caregivers’ QOL to ensure appropriate support can be provided [14].

While numerous instruments have been developed to measure cancer patients’ QOL, only a limited number are available for their caregivers [17,18]. Among these, Weitzner’s [19,20] Quality of Life Index-Cancer (CQOLC) is the most widely used. The CQOLC is a multidimensional tool consisting of 35 items across 4 main factors, with well-tested psychometric properties [19,20]. The original CQOLC has been validated in multiple languages, including American English [20], French, Turkish and Chinese [21–23]. Although a Malay version is available, to the best of our knowledge, it has only been validated among caregivers of specific diseases such as HIV [24] and gastrointestinal cancer [3]. To date, no validation studies have been conducted among caregivers of general cancer patients in Malaysia. This represents a significant gap, as the lack of a validated tool for the wider population of cancer caregivers limits the ability to carry out research, systematically monitor caregiver well-being, and develop targeted support programs. Given the substantial challenges faced by cancer caregivers, having a reliable and validated assessment instrument is essential for understanding their needs and improving their quality of life. Therefore, we initiated this study to validate the Malay version of the CQOLC among caregivers of cancer patients, including assessments of internal consistency, test-retest reliability, and confirmation of the original factor structure, to provide a robust tool for use in both clinical and research settings.

## Materials and methods

### Study design

This cross-sectional study involved primary caregivers of advanced cancer patients, who were sampled from the oncology clinic, wards and day care unit of Hospital Sultan Ismail, Johor Bahru (HSI JB) between 01-09-2022 to 31-07-2023 using convenience sampling. HSI JB is the primary government oncology centre in southern Peninsular Malaysia, providing comprehensive cancer care including follow-up consultations, chemotherapy and radiotherapy treatments. Primary caregivers were defined as individuals close to the patient who assumed responsibility for caring for them when the patient could no longer care for themselves. Participants were eligible for the study if they were 18 years or older, sufficiently literate in Bahasa Malaysia and able to provide informed consent. Exclusion criteria included individuals with a diagnosed mental illness and those unwilling to provide consent to participate in the study. Bujang et al. suggested for a minimum of five to seven respondents for each item in a questionnaire is required if the construct validity is fairly moderate with no other issues. Thus, a minimum of 310 samples were required with an additional 20% dropout rate [25].

### Ethical consideration

Approval from the Medical Research Ethics Committee, Ministry of Health Malaysia was obtained before the commencement of the study, approval number: NMRR ID-22-00780-L7O. Written informed consent was obtained from all participants who agreed to participate.

### Data collection procedures

Trained data collectors briefly explained the questionnaire to eligible participants from the target population after obtaining their consent. The MCQoL questionnaire took approximately 15-20 minutes to complete and participants returned their completed forms to the data collectors on the same day. To assess test-retest reliability, participants completed the MCQoL questionnaire again after a two-week interval, which falls within the recommended 1-4 week period for test-retest reliability assessment [26].

### Study instruments

#### Demographic and care-giving information.

A Case Report Form (CRF) was used to collect information on socio-demographic variables (gender, age, ethnicity, marital status, education level, household income, employment status, ability to manage with their current income), the caregiver’s relationship to the patient, care-related factors (the number of hours per day spent providing care, any shared care giving, past care giving experience) and chronic illness status.

#### Malay Caregiver Quality of Life (MCQoL).

The Malay Caregiver Quality of Life (MCQoL) is a translated version of the original English Caregiver Quality of Life Index-Cancer (CQOLC) scale [20]. The CQOLC scale was initially designed to assess the quality of life among Caucasian caregivers of cancer patients in Texas. It demonstrated strong reliability (Cronbach’s α = 0.95) and construct validity and has since been widely translated into various languages, including Malay [24]. The Malay version was originally validated among Malaysian family caregivers of HIV/AIDS patients and reported good reliability, with Cronbach’s α values ranging from 0.78 to 0.84 [24]. The instrument is a self-administered questionnaire designed to assess caregivers’ quality of life. It consists of 35 items divided into four domains: i) burden (10 items), ii) disruptiveness (7 items), iii) positive adaptation (7 items) and iv) financial concern (3 items). Additionally, there are eight single items addressing other factors, including disruption of sleep, satisfaction with sexual functioning, day-to-day focus, mental strain, informed about illness, protection of patient, management of patient’s pain and family interest in care giving. Each item is scored based on a 5-point Likert scale measurement: i) 0 - not at all, ii) 1 - a little bit, iii) 2 - somewhat, iv) 3 - quite a bit and v) 4 - very much. The total score is calculated by summing up the scores of all 35 items [27]. A higher total score reflects a worse quality of life (QoL) [27].

### Statistical analysis

Statistical analysis was performed using IBM SPSS Statistics for Windows, version 27.0 and IBM SPSS AMOS. A P-value of less than 0.05 was considered statistically significant. Descriptive statistics were used to summarize the socio-demographic characteristics of the participants. For continuous data, the mean and standard deviation (SD) were used, while frequency and percentages (%) were used for categorical data.

Exploratory Factor Analysis (EFA) using principal axis factoring (PAF) with varimax rotation was conducted to determine the construct validity of the MCQoL. An eigenvalue > 1.00 and the interpretation of the scree plot were considered for identifying the number of factors in the construct. The correlation between items was assessed using Bartlett’s test of sphericity, while sample adequacy was measured using the Kaiser-Meyer-Olkin (KMO) test. The MCQoL questionnaire was considered adequate if the KMO value exceeded 0.6. Bartlett’s test of sphericity was deemed significant if the p-value was less than 0.05. Items with factor loadings of ≥0.40 were considered to have an acceptable factor loading [28].

Confirmatory Factor Analysis (CFA) was then performed to test the fit constructs of the MCQoL. Model fit was evaluated using the following fit indices: a $\chi^{2}$/df ratio below 3, Comparative Fit Index (CFI) greater than 0.90, a Tucker-Lewis Index (TLI) above 0.90 and a Root Mean Square Error of Approximation (RMSEA) less than 0.08. [29–31]. Model revision was guided by areas of misfit, specifically modification indices more than 3.84, residual more than 2.58 and factor loading less than 0.50 [30]. Cronbach’s alpha coefficient and intra-class correlation (ICC) were used to assess internal consistency and test-retest stability, respectively. A Cronbach’s alpha value greater than 0.5 was considered acceptable, while a value exceeding 0.7 was regarded as good [32]. A corrected item-to-total correlation of more than 0.3 was deemed acceptable [33]. For ICC values, the following criteria were used: ≤0.40 as poor to fair, 0.41–0.60 as moderate, 0.61–0.80 as good and ≥ 0.80 as excellent [34,35].

## Results

### Demographic characteristics

The characteristics of the participants are presented in Table 1. A total of 309 caregivers (99.68 %) of cancer patients were recruited for the study. The mean age of the participants was 44.00 ± 14.22 years. The majority of participants were female (62.78 %), Malay (70.55 %), married (68.28 %) and had attained a secondary level of education (50.49 %). Spouses made up the largest group of caregivers (47.00 %), followed by children and parents of patients. On average, caregivers spent 75.50 ± 50.60 hours per week providing care.

**Table 1:** Participants’ socio-demographic characteristics (n = 309).

Characteristics	n	%
Age	44.00 (±14.22)	
**Gender**		
Male	115	37.22
Female	194	62.78
**Ethnicity**		
Malay	218	70.55
Chinese	73	23.62
Indian	15	4.85
Others	3	0.97
**Marital Status**		
Single	92	29.87
Married	211	68.28
Widow/Divorced	6	1.94
**Education**		
No Formal Education	16	5.18
PMR	19	6.15
SPM	137	44.34
Diploma	75	24.27
Ijazah/Master/PhD	62	20.06
**Employment Status**		
Retired	19	6.15
Employed	178	57.61
Unemployed	44	14.24
Housewife	67	21.68
**Living with Patient**		
Yes	242	78.32
No	67	21.68
**Shared Care-giving**		
Yes	200	64.72
No	108	34.95

### Construct validity

This study reported a sampling adequacy of 0.90 from the Kaiser-Meyer-Olkin (KMO) measure and a significant Bartlett’s test of sphericity (p < 0.001). Exploratory Factor Analysis (EFA) using principle axis factoring with varimax rotation indicated that the MCQoL was valid. Varimax rotation was used because the constructs were uncorrelated and the rotation minimizes the number of items with higher factor loadings on each construct [27,28]. A total of four factors were extracted for the MCQoL, with eigenvalues ranging from 1.46 to 9.01, accounting for a total variance of 53.81%. Items with factor loadings of less than 0.40 were removed from the construct, as shown in Table 2. The three items removed were item numbers 4, 5, and 35, which corresponded to satisfaction with sexual functioning, maintenance of outside activities and family interest in caregiving, respectively.

**Table 2:** Exploratory Factor Analysis of MCQoL (4-factor model).

Item	F1	F2	F3	F4	1*Original
Q1: Alteration in daily routine	0.74				Disruptiveness
Q2: Disruption of sleep	0.60				-
Q3: Impact on daily schedule	0.76				Disruptiveness
Q13: Day-to-day focus	0.65				-
Q24: Transportation	0.68				Disruptiveness
Q26: Responsibility for patient’s care	0.71				Disruptiveness
Q29: Change in priorities	0.76				Disruptiveness
Q30: Protection of patient	0.79				-
Q32: Management of patient’s pain	0.78				-
Q9: Death of patient		0.44			Burden
Q11: Level of stress		0.54			Burden
Q14: Sadness		0.65			Burden
Q15: Mental Strain		0.54			-
Q17: Guilt		0.68			Burden
Q18: Frustration		0.68			Burden
Q19: Nervousness		0.69			Burden
Q20: Impact of illness on family		0.54			Burden
Q21: Patient’s eating habits		0.53			Disruptiveness
Q25: Adverse effects of treatment		0.54			Burden
Q31: Deterioration of patient		0.53			Burden
Q33: Future outlook		0.53			Burden
Q10: Outlook on life			0.60		Positive Adaptation
Q12: Spirituality			0.64		Positive Adaptation
Q16: Social support			0.59		Positive Adaptation
Q22: Relationship with patient			0.78		Positive Adaptation
Q23: Informed about illness			0.66		-
Q27: Focus of caregiving			0.77		Positive Adaptation
Q28: Family communication			0.78		Positive Adaptation
Q34: Family support			0.74		Positive Adaptation
Q6: Financial strain				0.84	Financial Concern
Q7: Concern about insurance				0.58	Financial Concern
Q8: Economic future				0.81	Financial Concern
Eigenvalue	9.01	4.86	1.89	1.46	
Variance, %	17.66	14.35	14.26	7.53	
Cumulative variance, %	17.66	32.02	46.28	53.81	

EFA models were refitted after exclusion of the 3 items (S4, S5 and S35) in light of Factor Loadingeak < 0.40. Varimax rotated of Principle Component Analysis; KMO value = 0.90; Barlett’s Test of Sphericity p < 0.001.

The EFA revealed that the Disruptiveness domain contained 9 items, with factor loadings for items 1, 2, 3, 13, 24, 26, 29, 30 and 32 ranging from 0.60 to 0.78. The Burden domain included 12 items, namely items 9, 11, 14, 15, 17, 18, 19, 20, 21, 25, 31 and 33 with factor loadings ranging from 0.44 to 0.69. The Positive Adaptation domain consisted of 8 items, with factor loadings for items 10, 12, 16, 22, 23, 27, 28 and 34 ranging from 0.59 to 0.78. Lastly, the Financial Concern domain had 3 items, with factor loadings for items 6, 7 and 8 ranging from 0.58 to 0.84. A summary of the exploratory factor analysis of the MCQoL is presented in Table 2.

**Fig 1:**
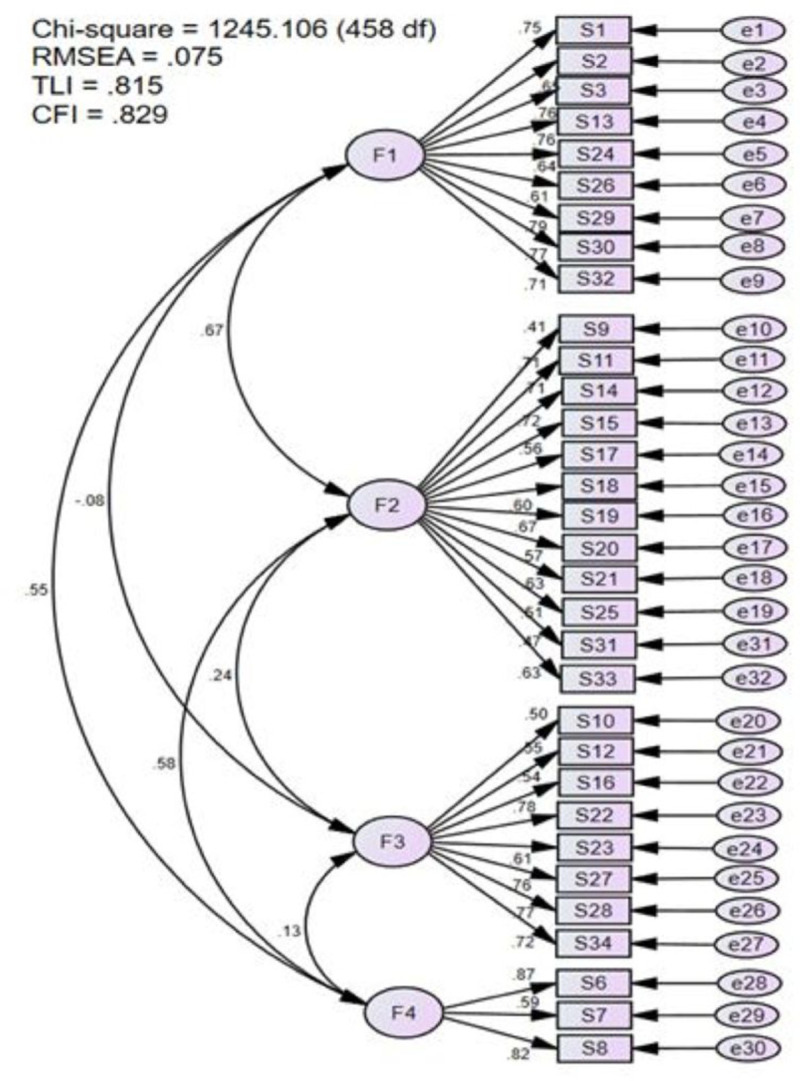
Model 1: Confirmatory Factor Analysis of MCQoL (4 factor model with 32 items).

**Fig 2:**
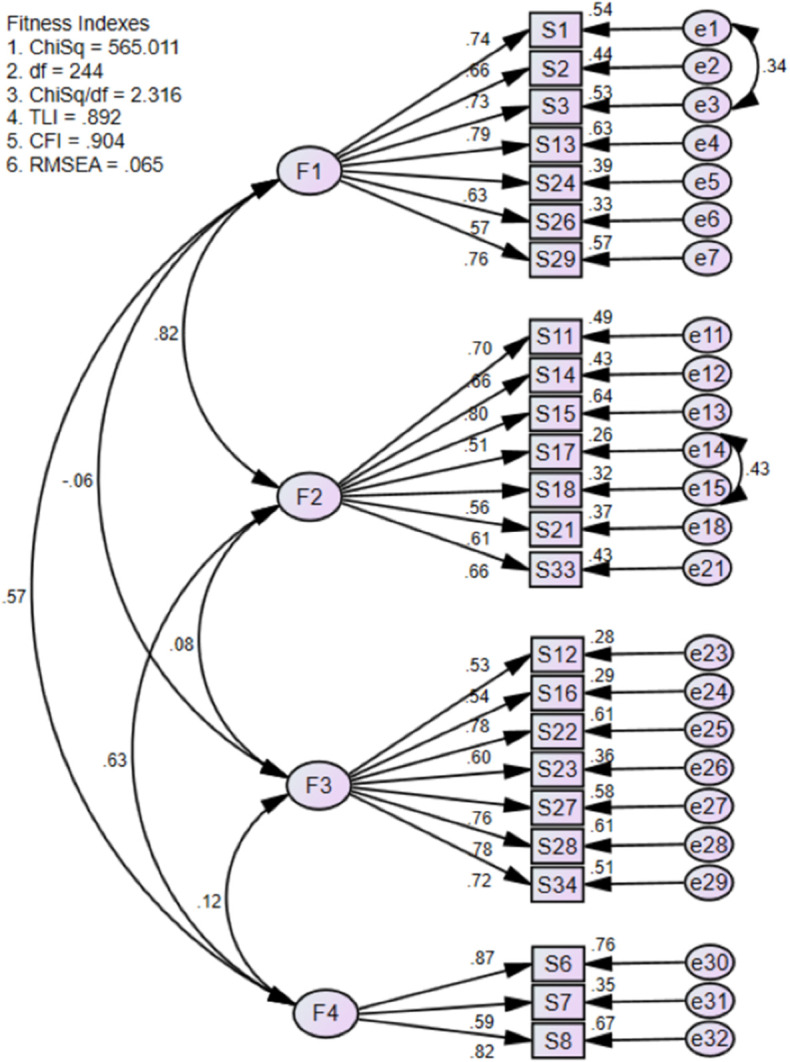
Final model: Confirmatory Factor Analysis of MCQoL (4 factor model with 24 items).

We performed a model 1 of CFA of the 4-factor model of the MCQoL questionnaire, which allocated items to domains based on the findings from the EFA of this study. Specifically, items 1, 2, 3, 13, 24, 26, 29, 30 and 32 were assigned to the Disruptiveness domain; items 9, 11, 14, 15, 17, 18, 19, 20, 21 and 25 were allocated to the Burden domain; items 10, 12, 16, 22, 23, 27, 28 and 34 were designated for the Positive Adaptation domain; and items 6 to 8 were allocated to the Financial Concern domain. The model 1 of CFA of the MCQoL questionnaire revealed that the 4-factor model with 32 items did not fit the data well, with a chi-square value of p < 0.05, CFI = 0.829, TLI = 0.815, and RMSEA = 0.075 (Fig 1). Several revisions to the CFA model were undertaken to address areas of misfit. Specifically, items with modification indices greater than 3.84, standardized residuals above 2.58 and factor loadings below 0.50 were considered problematic. Problematic items were removed, or, where theoretically appropriate, correlating error between two items was applied. In total, eight items (numbers 9, 10, 19, 20, 25, 30, 31 and 32) were removed, while two pairs of error terms for items within the same factor were allowed to correlate. The final CFA model, comprising 24 items across four factors, demonstrated an acceptable fit: RMSEA criterion = 0.065, CFI= 0.904, Chisq/df ($\chi^{2}$/df ratio) = 2.316 and moderate fit for TLI = 0.892. The final CFA model, presented in Figure 2, identified four distinct domains. The Disruptiveness domain comprised seven items (1, 2, 3, 13, 24, 26 and 29), with factor loadings ranging from 0.57 to 0.76. The Burden domain also included seven items, specifically, items 11, 14, 15, 17, 18, 21 and 33, with factor loadings between 0.51 and 0.80. The Positive Adaptation domain consisted of eight items (10, 12, 16, 22, 23, 27, 28 and 34), with loadings spanning 0.53 to 0.78. Finally, the Financial Concern domain contained three items (6, 7 and 8), with factor loadings ranging from 0.59 to 0.87 (Fig 2).

### Reliability

To assess the reliability of the final model of the MCQoL questionnaire, internal consistency was evaluated using Cronbach’s alpha coefficient (Table 3). The overall Cronbach’s alpha value for the MCQoL questionnaire was 0.88. All sub-scales demonstrated relatively high Cronbach’s alpha values, ranging from α = 0.80 to 0.87. Additionally, test-retest stability was assessed as another measure of reliability. The intraclass correlation coefficient (ICC) for all constructs indicated fair to good stability, with values ranging from 0.37 to 0.77.

**Table 3:** Reliability of final Confirmatory Factor Analysis of MCQoL (4 factor model with 24 items).

Factor	Number of items	Cronbach’s alpha (α)	^*a*^ICC
F1: Disruptiveness	7	0.87	0.72
F2: Burden	7	0.84	0.71
F3: Positive Adaptation	7	0.85	0.37
F4: Financial concern	3	0.80	0.64
Overall	24	0.88	0.77

^*a*^Test-retest: ICC-Intra-class correlation.

## Discussion

This study aimed to examine the validity and reliability of the Malay version of the CQoL (MCQoL). The original MCQoL is an adapted scale derived from the English CQOLC, an instrument designed to measure the disease-specific quality of life of cancer patients’ family members or caregivers. We obtained several key and novel findings. The final MCQoL demonstrated excellent internal consistency, with a Cronbach’s alpha of 0.88, which comparable to the original English CQOLC (α = 0.91) [20] and surpasses the reliability previously reported for Malaysian HIV/AIDS caregivers (α = 0.73 - 0.84) [24]. The alignment between the final model of MCQoL and the original English CQOLC likely stems from the instrument’s specific focus on cancer caregiving, whereas the lower reliability among HIV/AIDS caregivers may reflect differences in population and care context.

In terms of scale validity, EFA identified four factors for the final MCQoL, consistent with both the original MCQoL and the English CQOLC. Three items (4, 5, and 35) were removed due to low factor loadings of below 0.40 [28]. The elimination of Item 4, “Satisfaction with sexual functioning,” reflects deep-rooted cultural sensitivities in Malaysian society, where open discussions about intimate matters remain taboo, potentially affecting response validity. Similarly, the removal of Item 5, “Maintenance of outside activity” highlights cultural differences, particularly the Malaysian emphasis on collective family welfare over individual pursuits. In this context, family priorities often supersede personal interests, especially when caring for a loved one with cancer. If a patient resists treatment, relatives may appeal to familial obligations, encouraging them to “think about your parents or children.” In such life-and-death situations, discussing personal activities outside the family is often considered inappropriate or even selfish [36].

Although the factor structure of the MCQoL differs from international adaptations such as the Chinese and German versions, these variations likely reflect authentic cultural distinctions rather than measurement flaws. The redistribution of items across domains suggests that the conceptualization of caregiver quality of life is shaped by socio-cultural values, particularly in cultures with strong familial orientations. This underscores the importance of cultural adaptation in psychometric instrument development, highlighting the importance of addressing not only linguistic translation but also deeper cultural contexts that influence caregiving experiences and their assessment.

Interestingly, the items in the final MCQoL EFA model closely aligned with the subscales of the original MCQoL and the English CQOLC model [20]. The first factor included 9 out of the 32 items, with five items (1, 3, 24, 26 and 29) mapping directly to the “Disruptiveness” subscale in the original MCQoL and English CQOLC versions. Four additional items (2, 13, 30 and 32), not originally included in any subscale, also loaded onto this factor [20,24]. The second factor consisted of twelve items, all ten (9, 11, 14, 17, 18, 19, 20, 25, 31 and 33) from the “Burden” subscale of the earlier models, as well as item 21 (from “Disruptiveness”) and item 15 (previously unassigned). The third factor comprised of all seven items (10, 12, 16, 22, 27, 28 and 34) from the “Positive Adaptation” subscale of the original models, along with one additional item (23) (previously unassigned). Finally, the fourth factor included all 3 items (6, 7, and 8) from the “Financial Concern” subscale in the English CQOLC version [8], although this differed slightly from the original MCQoL, where only items 6 and 8 loaded in this domain [24]. This factor structure highlights both the conceptual consistency with theoretical models and meaningful refinement through cultural adaptation, ensuring the final MCQoL’s relevance to Malaysian caregiving experiences.

Noted that the initial CFA model 1 fit indices for MCQoL were mixed. While the TLI and CFI values were outside the acceptable range, the $\chi^{2}$/df ratio and RMSEA met criteria for acceptable fit. To improve the CFA model 1, eight problematic items were removed. Four items were removed due to low factor loadings (items 9, 10, 20 and 31) and high residuals (items 19 and 25). Additionally, two items (30 and 32) were removed based on high modification indices. This approach aligned with the English CQOLC version, where items 30 and 32 were similarly not included in subscales [20]. Besides, two pairs of items within the same factors were allowed to have correlated errors in the model due to high modification indices. These correlations were theoretically justified and consistent with the original English CQOLC version [20].

In the final model, the first factor (Disruptiveness) included seven out of the twenty-four items: five items (1, 3, 24, 26 and 29) matched the original MCQoL and English CQOLC versions [20,24], and two items (2 and 13) were not previously assigned to specific subscales. The second factor (Burden) consisted of seven items, including five (11, 14, 17, 18 and 33) aligning with the original MCQoL and English CQOLC versions [20,24], one item (21) from “Disruptiveness” and another item (15) not originally assigned. The third factor (Positive Adaptation) comprised six items (10, 12, 16, 22, 27, 28 and 34) from the original models, plus one additional item (23) that did not align with the original four subscales. Finally, the fourth factor (Financial Concern) included all three items (6, 7 and 8) from the English CQOLC version [20].

The model fit indices for the final four-factor MCQoL, containing 24 items, indicated an acceptable fit. Specifically, the $\chi^{2}$/df ratio was 2.32 (below the threshold of 3), the RMSEA was 0.06 (less than 0.08), and the CFI was 0.90 (above 0.90), demonstrating good alignment between the hypothesized structure and the sample data [37]. The CFA results confirmed the multidimensional nature of the MCQoL instrument. Notably, CFA was not performed for the original MCQoL or the English version of the CQOLC, while the German adaptation’s CFA reported poor model fit indices despite item removals [38]. These findings highlight the inherent challenges of achieving optimal model fit in cross-cultural validations, as differences in item interpretation and cultural context can affect factor structures. This study has several limitations that should be acknowledged. First, the use of single-center sampling restricts the generalizability of the findings, as participants were drawn from only one institution and may not represent the broader population. Second, convenience sampling was employed, which can introduce selection bias and limit the representativeness of the sample. However, this is the first study to date to validate the MCQoL questionnaire for caregivers of cancer patients in Malaysia. Therefore, it serves as an important stepping stone for future research, providing a foundation for larger-scale, multi-centre studies that can further confirm and extend these findings.

## Conclusion

The final MCQoL scale demonstrated acceptable reliability and validity findings. The four-factor MCQoL model achieved a cumulative explained variance of 53.81%, and the internal consistency reliability was good for both the overall scale and its four domains. Based on these findings, the final MCQoL can be considered a reliable and valid instrument for assessing the quality of life of cancer caregivers in Malaysia. This scale holds potential for use by researchers and healthcare practitioners to better understand and address the challenges faced by this population.
